# Correlation of the oxygen radical activity and antioxidants and severity in critically ill surgical patients: preliminary report

**DOI:** 10.1186/cc11943

**Published:** 2013-03-19

**Authors:** J Lee, H Shim, JY Jang

**Affiliations:** 1 Yonsei University College of Medicine, Seoul, South Korea; 2 Wonju Severance Christian Hospital, Yonsei University Wonju College of Medicine, Wonju, Korea

## Introduction

In septic patients, the oxygen radical (OR) showed toxic effect to induce inflammation and antioxidant activity could affect organ dysfunction. This study was designed to determine the relationship between antioxidant level and severity of organ dysfunction.

## Methods

The medical records of adult patients managed in a surgical ICU from August 2012 to December 2012 were reviewed prospectively. Abstracted data included age, body weight (with BMI), APACHE II scores, SOFA scores, MODS scores, fluid intake, fluid output, nutritional support, shock, antioxidant levels, OR activities, zinc and selenium levels, complication and mortality. In addition, length of stay (LOS) in the ICU and in hospital, and in-hospital mortality were collected. These data were investigated on the first, the third and the seventh day, respectively.

## Results

A total of 13 patients were enrolled. The in-hospital mortality rate was 7.7% and mean LOS in the ICU and hospital was 6.5 and 27.6, respectively. Mean APACHE II score was 20.2. On the first day of ICU, the mean antioxidant level and OR were 1.5 (± 0.5) mmol/l and 1.6 (± 0.5) mmol/l, respectively. At the same time, SOFA and MODS scores were 7.3 and 5.0, respectively, and zinc and selenium were 32.6 µg/dl and 68.4 ng/ml. On the third day, mean antioxidant and OR were 1.5 (± 0.4) and 1.8 (± 0.7) respectively (SOFA 6.6, MODS 4.9, zinc 50.0, selenium 70.7). On the seventh day, mean antioxidant and OR were 1.4 (± 0.5) and 1.9 (± 0.7), respectively (SOFA 4.3, MODS 3.1, zinc 62.8, selenium 77.3). In the correlation analysis, MODS scores and antioxidant level had significant correlations on the first and seventh days of ICU (*P *= 0.001, *P *= 0.009).

## Conclusion

Antioxidant level had a correlation with organ dysfunction which might be used as a prognostic factor in critically septic patients. To prove this, large-scale data collection is required.
